# Supplementary material for: Human Holliday junction resolvase GEN1 uses a chromodomain for efficient DNA recognition and cleavage
Source: eLife. 2015 Dec 18;4:e12256. doi: 10.7554/eLife.12256 (PMC5039027; doi:10.7554/eLife.12256)
Supplement: Figure 3—source data 1. — Top hits found in a DALI search for protein structure comparison with the human GEN1 chromodomain (residues 390–464) against the Protein Data Bank. The most similar unique chromodomains are listed. DOI: http://dx.doi.org/10.7554/eLife.12256.008 [file elife-12256-fig3-data1.docx]

| **Protein** | **Organism** | **PDB code** | **Z-Score**  **(Dali)** | **RMSD**  **(Dali)** |
| --- | --- | --- | --- | --- |
| Heterochromatin protein 1 (HP1) | Drosophila melanogaster | 3p7j | 8.1 | 2.8 |
| M-phase phosphoprotein 8 (MPP8) | Homo sapiens | 3r93 | 7.9 | 2.1 |
| Rhino (HP1 homolog) | Drosophila melanogaster | 4quf | 7.8 | 2.0 |
| Chromobox protein homolog 7 (CBX7) | Mus musculus | 4x3s | 7.4 | 2.3 |
| Chromobox protein homolog 3 (CBX3) | Homo sapiens | 3kup | 7.3 | 1.8 |
| Chromobox protein homolog 1 (CBX1) | Homo sapiens | 3q6s | 7.1 | 1.9 |
| Suppressor of variegation 3-9 homolog 1 (SUV39H1) | Homo sapiens | 3mts | 7.1 | 2.2 |
| Chromobox protein homolog 6 (CBX6) | Homo sapiens | 3gv6 | 6.8 | 2.3 |
| Polycomb (Pc) | Drosophila melanogaster | 1pfb | 6.6 | 2.5 |
| Chromobox protein homolog 8 (CBX8) | Homo sapiens | 3i91 | 6.6 | 2.1 |
