# Supplementary material for: Human Holliday junction resolvase GEN1 uses a chromodomain for efficient DNA recognition and cleavage
Source: eLife. 2015 Dec 18;4:e12256. doi: 10.7554/eLife.12256 (PMC5039027; doi:10.7554/eLife.12256)
Supplement: Figure 3—source data 2. — DOI: http://dx.doi.org/10.7554/eLife.12256.009 [file elife-12256-fig3-data2.docx]

| **Peptide** | **Sequence (N->C)** |
| --- | --- |
| H3K9 | ARTKQTARKSTGGKA |
| H3K9me1 | ARTKQTAR-K(Me1)-STGGKA |
| H3K9me2 | ARTKQTAR-K(Me2)-STGGKA |
| H3K9me3 | ARTKQTAR-K(Me3)-STGGKA |
| H3K27 | QLATKAARKSAPATGGV |
| H3K27me1 | QLATKAAR-K(Me1)-SAPATGGV |
| H3K27me2 | QLATKAAR-K(Me2)-SAPATGGV |
| H3K27me3 | QLATKAAR-K(Me3)-SAPATGGV |
| H3K36me1 | SAPATGGV-K(Me1)-KPHRYRP |
| H3K36me2 | SAPATGGV-K(Me2)-KPHRYRP |
| H3K36me3 | SAPATGGV-K(Me3)-KPHRYRP |
| H3K36Ac | SAPATGGV-K(Ac)-KPHRYRP |
| H4K20me3 | KGGAKRHR-K(Me3)-VLRDNIQ |
