# Supplementary material for: Human Holliday junction resolvase GEN1 uses a chromodomain for efficient DNA recognition and cleavage
Source: eLife. 2015 Dec 18;4:e12256. doi: 10.7554/eLife.12256 (PMC5039027; doi:10.7554/eLife.12256)
Supplement: Figure 5—source data 1. — Four-way junctions were prepared by annealing CB209, CB210, CB211, CB212. 5’ flaps were prepared by annealing CB209, CB212, and CB218. The annealing protocol is described in Material and methods. DOI: http://dx.doi.org/10.7554/eLife.12256.014 [file elife-12256-fig5-data1.docx]

**Oligonucleotides used in biochemical assays.**

| **Oligonucleotide** | **Sequence (5’->3’)** |
| --- | --- |
| CB209(5’ 6FAM) | ACGCTGCCGAATTCTACCAGTGCCTTGCTAGGACATCTTTGCCCACCTGCAGGTTCACCC |
| CB210 | GGGTGAACCTGCAGGTGGGCAAAGATGTCCATCTGTTGTAATCGTCAAGCTTTATGCCGT |
| CB211 | ACGGCATAAAGCTTGACGATTACAACAGATCATGGAGCTGTCTAGAGGATCCGACTATCG |
| CB212 | CGATAGTCGGATCCTCTAGACAGCTCCATGTAGCAAGGCACTGGTAGAATTCGGCAGCGT |
| CB218 | GGGTGAACCTGCAGGTGGGCAAAGATGTCC |
