# Supplementary material for: Human Holliday junction resolvase GEN1 uses a chromodomain for efficient DNA recognition and cleavage
Source: eLife. 2015 Dec 18;4:e12256. doi: 10.7554/eLife.12256 (PMC5039027; doi:10.7554/eLife.12256)
Supplement: Figure 5—source data 2. — DOI: http://dx.doi.org/10.7554/eLife.12256.015 [file elife-12256-fig5-data2.docx]

| **Strain** | **Full genotype** | **Relevant genotype** | **Source** |
| --- | --- | --- | --- |
| YDG291 | YEN1:hphNT1 | yen1∆ | This study |
| YLP170 | MUS81::KanMx | mus81∆ | This study |
| YLP171 | YEN1::hphNT1 MUS81::KanMx | mus81∆ yen1∆ | This study |
| YLP203  (YLP289) | YEN1:hphNT1 leu2-3,112::pRS305-YEN1::LEU2 | yen1∆ YEN1 | This study |
| YLP204  (YLP290) | YEN1:hphNT1 leu2-3,112::pRS305-YEN1-F478A::LEU2 | yen1∆ YEN1-F478A | This study |
| YLP208  (YLP292) | YEN1:hphNT1 leu2-3,112::pRS305-YEN1-W529A::LEU2 | yen1∆ YEN1-W529A | This study |
| YLP209  (YLP293) | YEN1:hphNT1 leu2-3,112::pRS305-YEN1-L530A::LEU2 | yen1∆ YEN1-L530A | This study |
| YLP211  (YLP294) | YEN1:hphNT1 leu2-3,112::pRS305-YEN1-K484E,R486E::LEU2 | yen1∆ YEN1-K484E,R486E | This study |
| YLP212  (YLP295) | YEN1:hphNT1 leu2-3,112::pRS305-YEN1-R486E::LEU2 | yen1∆ YEN1-R486E | This study |
| YLP213  (YLP296) | YEN1:hphNT1 leu2-3,112::pRS305-YEN1-K484E::LEU2 | yen1∆ YEN1-K484E | This study |
| YLP214  (YLP297) | YEN1:hphNT1 leu2-3,112::pRS305-YEN1-D41N::LEU2 | yen1∆ YEN1-D41N | This study |
| YLP215  (YLP298) | YEN1:hphNT1 leu2-3,112::pRS305-YEN1-F47E::LEU2 | yen1∆ YEN1-F47E | This study |
| YLP216  (YLP299) | YEN1:hphNT1 leu2-3,112::pRS305-YEN1-I97E::LEU2 | yen1∆ YEN1-I97E | This study |
| YLP217  (YLP300) | YEN1:hphNT1 leu2-3,112::pRS305-YEN1-K298E::LEU2 | yen1∆ YEN1-K298E | This study |
| YLP268  (YLP301) | YEN1:hphNT1 leu2-3,112::pRS305-YEN1-K469E::LEU2 | yen1∆ YEN1-K469E | This study |
| YLP269  (YLP302) | YEN1:hphNT1 leu2-3,112::pRS305-YEN1-Y487A::LEU2 | yen1∆ YEN1-Y487A | This study |
| YLP270  (YLP303) | YEN1:hphNT1 leu2-3,112::pRS305-YEN1-N526A::LEU2 | yen1∆ YEN1-N526A | This study |
| YLP271  (YLP304) | YEN1:hphNT1 leu2-3,112::pRS305-YEN1-L528D::LEU2 | yen1∆ YEN1-L528D | This study |
| YLP219  (YLP305) | YEN1:hphNT1 leu2-3,112::pRS305-YEN1::LEU2 MUS81::KanMx | mus81∆ yen1∆ YEN1 | This study |
| YLP220  (YLP306) | YEN1:hphNT1 leu2-3,112::pRS305-YEN1-F478A::LEU2 MUS81::KanMx | mus81∆ yen1∆ YEN1-F478A | This study |
| YLP224  (YLP308) | YEN1:hphNT1 leu2-3,112::pRS305-YEN1-W529A::LEU2 MUS81::KanMx | mus81∆ yen1∆ YEN1-W529A | This study |
| YLP225  (YLP309) | YEN1:hphNT1 leu2-3,112::pRS305-YEN1-L530A::LEU2 MUS81::KanMx | mus81∆ yen1∆ YEN1-L530A | This study |
| YLP227  (YLP310) | YEN1:hphNT1 leu2-3,112::pRS305-YEN1-K484E,R486E::LEU2 MUS81::KanMx | mus81∆ yen1∆ YEN1-K484E,R486E | This study |
| YLP228  (YLP311) | YEN1:hphNT1 leu2-3,112::pRS305-YEN1-R486E::LEU2 MUS81::KanMx | yen1∆ YEN1-R486E | This study |
| YLP229  (YLP312) | YEN1:hphNT1 leu2-3,112::pRS305-YEN1-K484E::LEU2 MUS81::KanMx | mus81∆ yen1∆ YEN1-K484E | This study |
| YLP230  (YLP313) | YEN1:hphNT1 leu2-3,112::pRS305-YEN1-D41N::LEU2 MUS81::KanMx | mus81∆ yen1∆ YEN1-D41N | This study |
| YLP231  (YLP314) | YEN1:hphNT1 leu2-3,112::pRS305-YEN1-F47E::LEU2 MUS81::KanMx | mus81∆ yen1∆ YEN1-F47E | This study |
| YLP232  (YLP315) | YEN1:hphNT1 leu2-3,112::pRS305-YEN1-I97E::LEU2 MUS81::KanMx | mus81∆ yen1∆ YEN1-I97E | This study |
| YLP233  (YLP316) | YEN1:hphNT1 leu2-3,112::pRS305-YEN1-K298E::LEU2 MUS81::KanMx | mus81∆ yen1∆ YEN1-K298E | This study |
| YLP272  (YLP317) | YEN1:hphNT1 leu2-3,112::pRS305-YEN1-K469E::LEU2 MUS81::KanMx | mus81∆ yen1∆ YEN1-K469E | This study |
| YLP273  (YLP318) | YEN1:hphNT1 leu2-3,112::pRS305-YEN1-Y487A::LEU2 MUS81::KanMx | mus81∆ yen1∆ YEN1-Y487A | This study |
| YLP274  (YLP319) | YEN1:hphNT1 leu2-3,112::pRS305-YEN1-N526A::LEU2 MUS81::KanMx | mus81∆ yen1∆ YEN1-N526A | This study |
| YLP275  (YLP320) | YEN1:hphNT1 leu2-3,112::pRS305-YEN1-L528D::LEU2 MUS81::KanMx | mus81∆ yen1∆ YEN1-L528D | This study |

All strains are based on W303 RAD5+: MAT**a** Rad5+ ade2 ura3-1 trp1-1 leu2-3,112 his3-11,15 can1-100. Strain numbers in brackets represent corresponding strains containing a 3FLAG-tagged version of YEN1 (YEN1-3FLAG::HIS3MX6).
